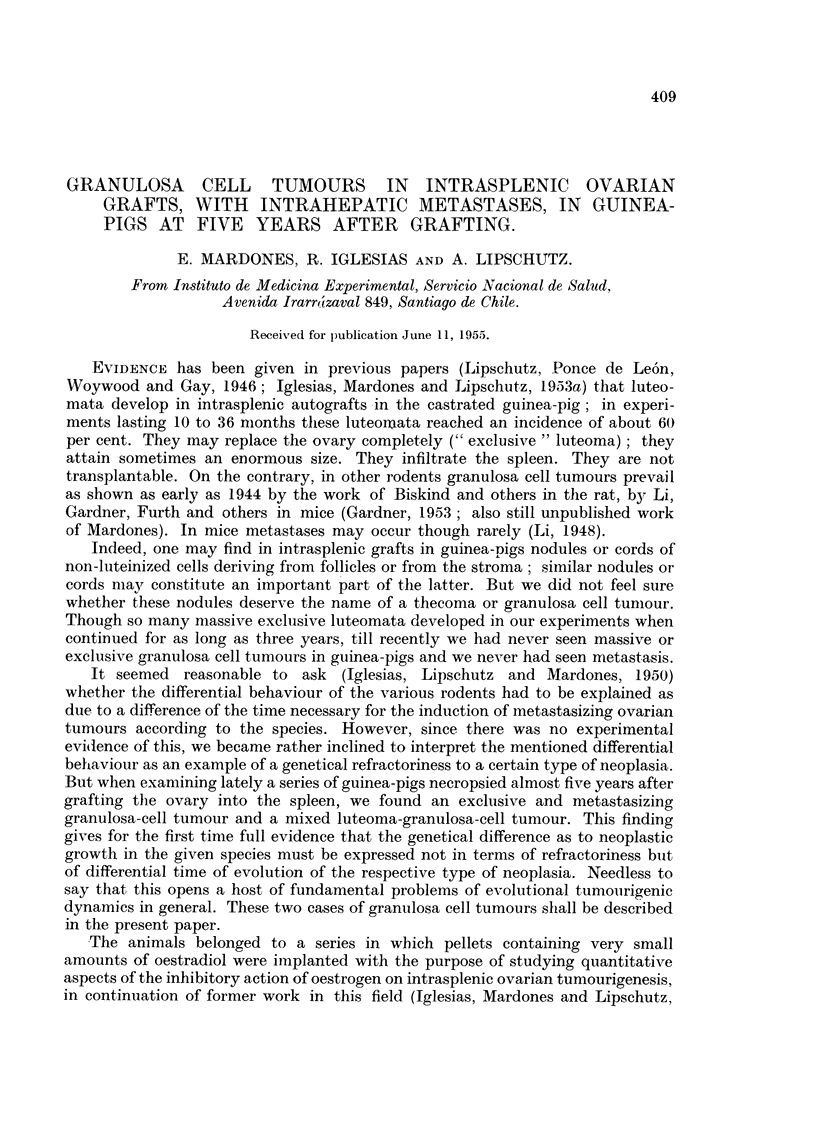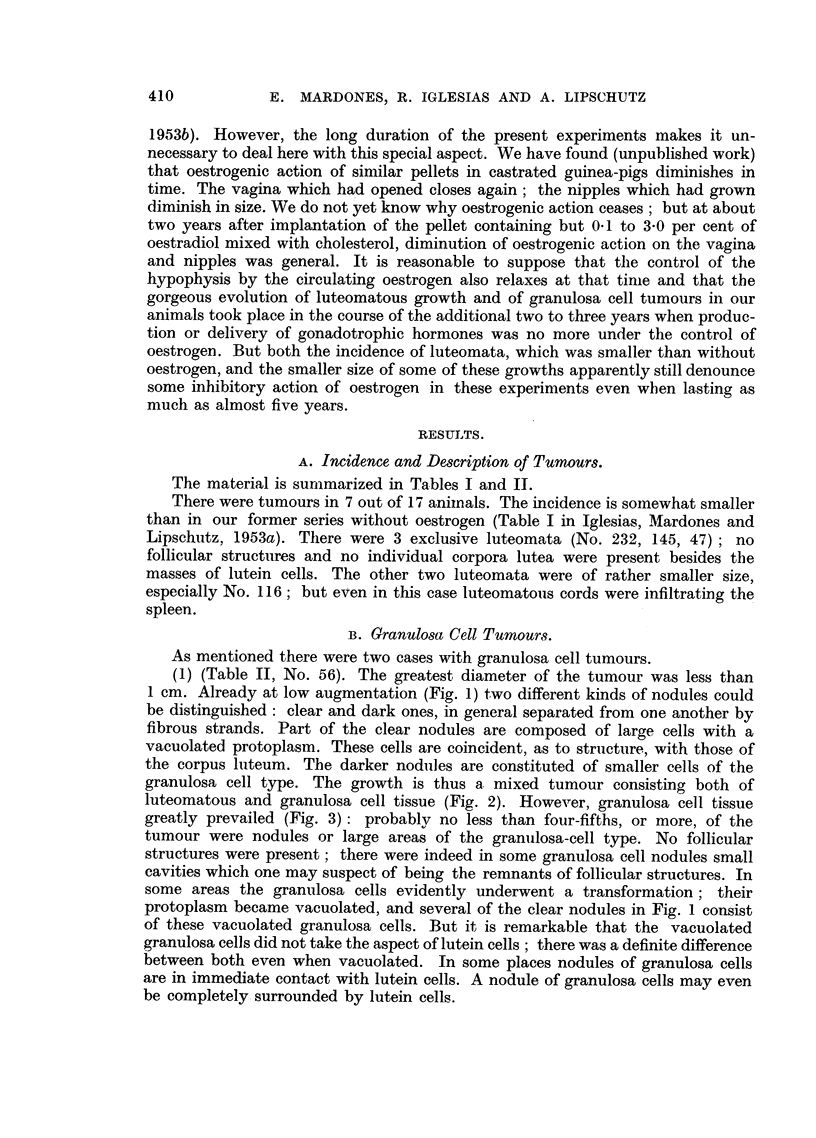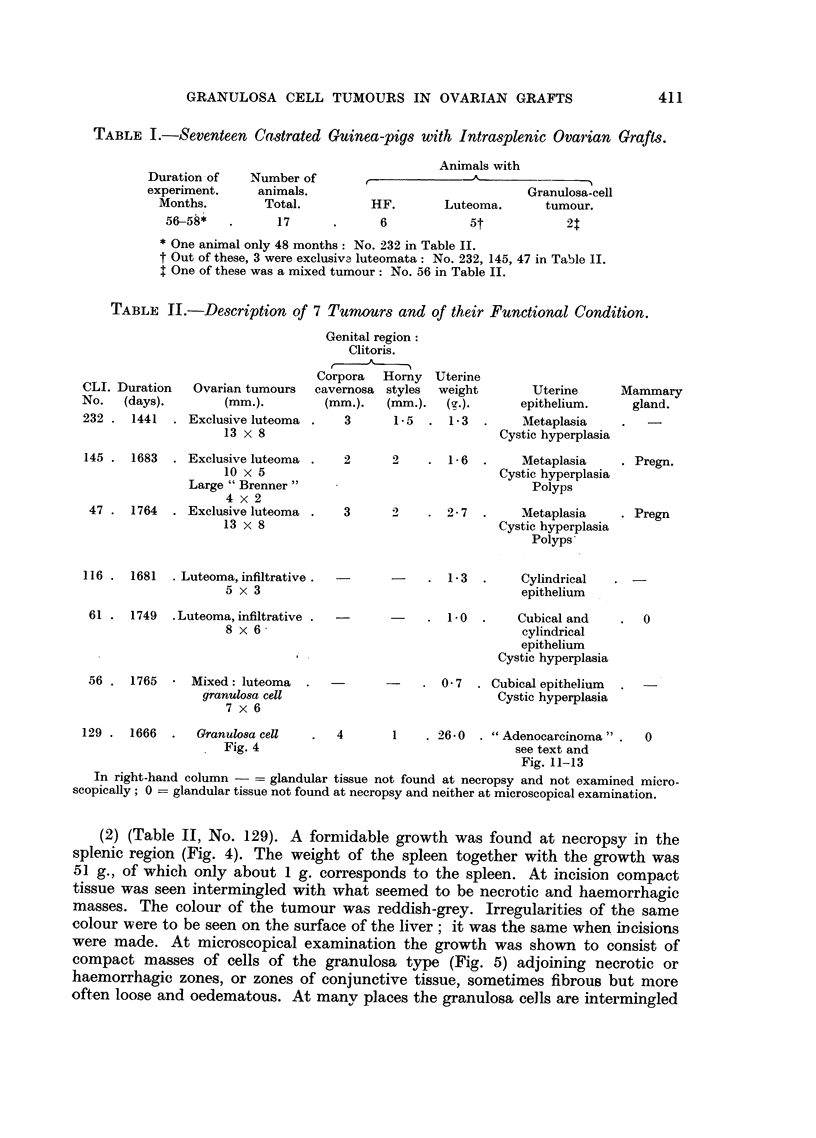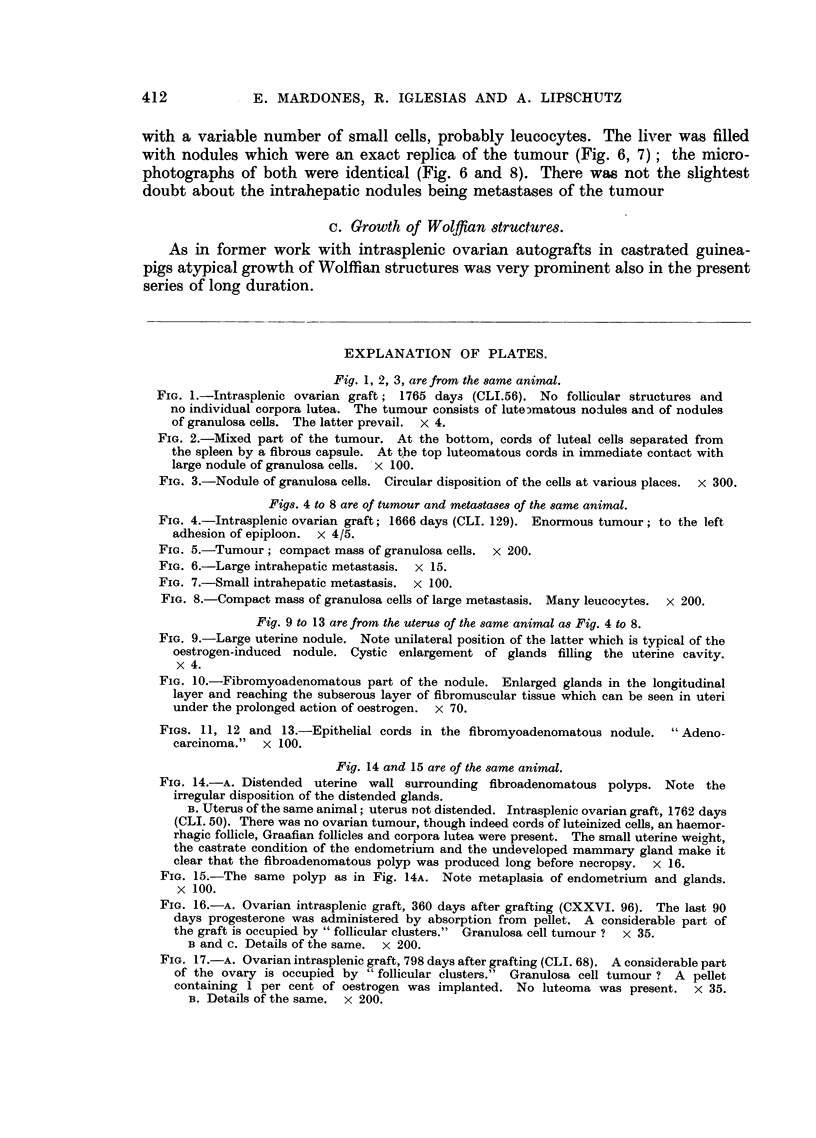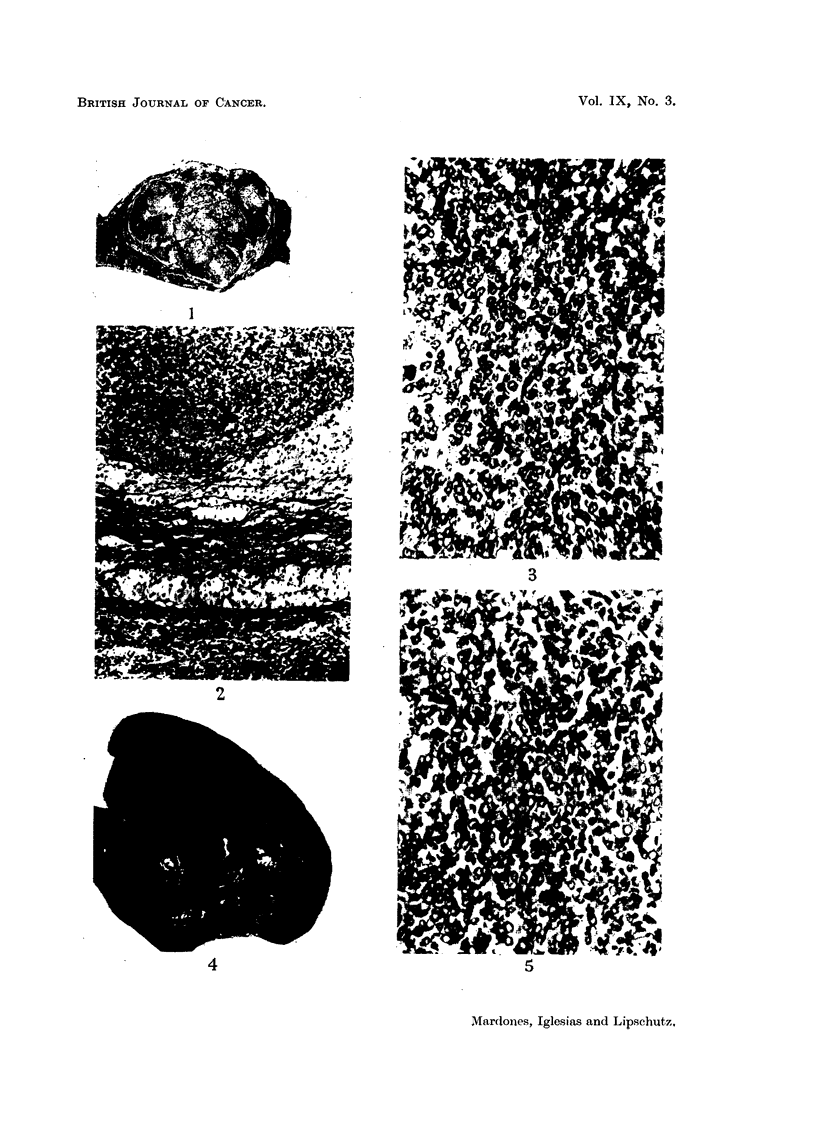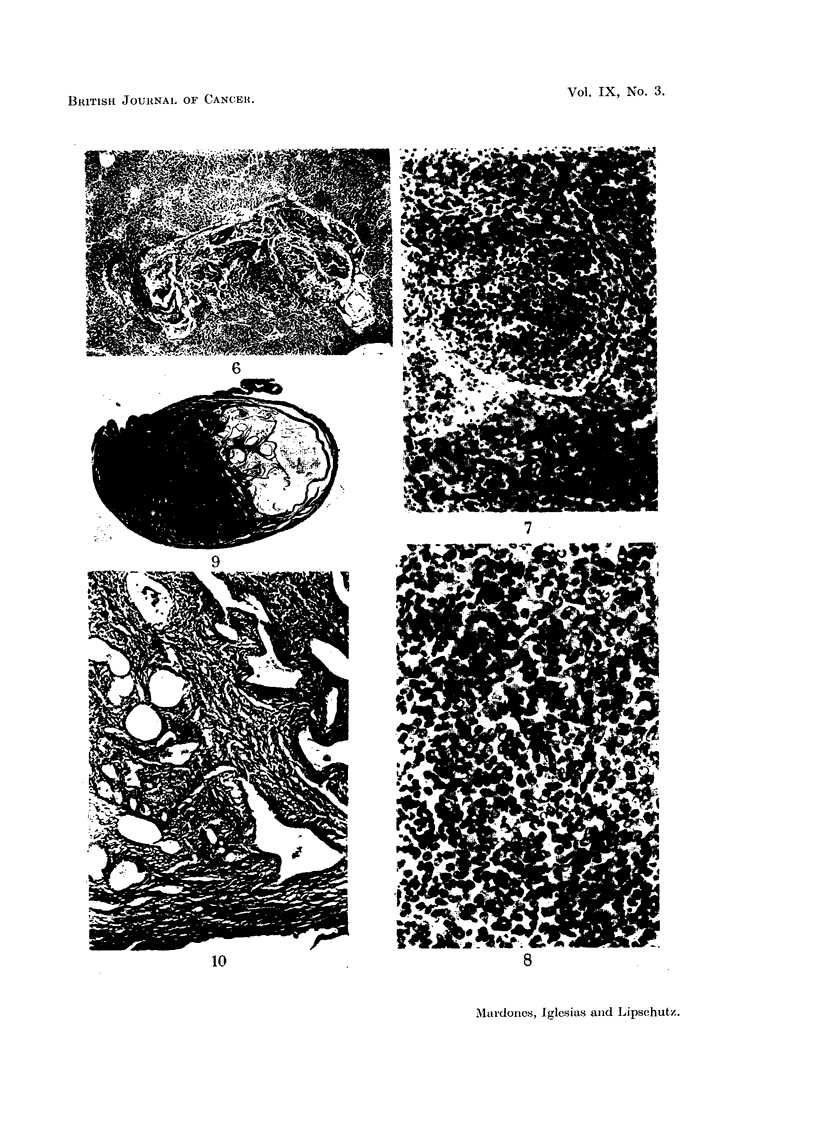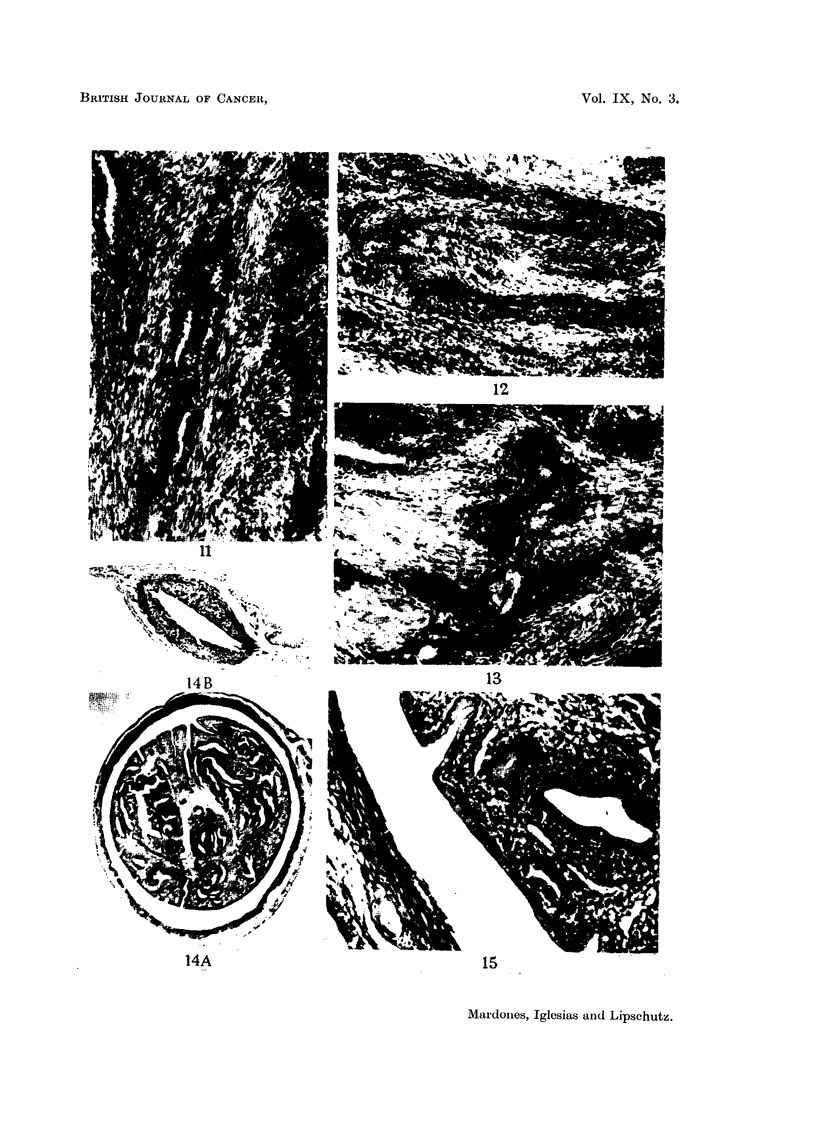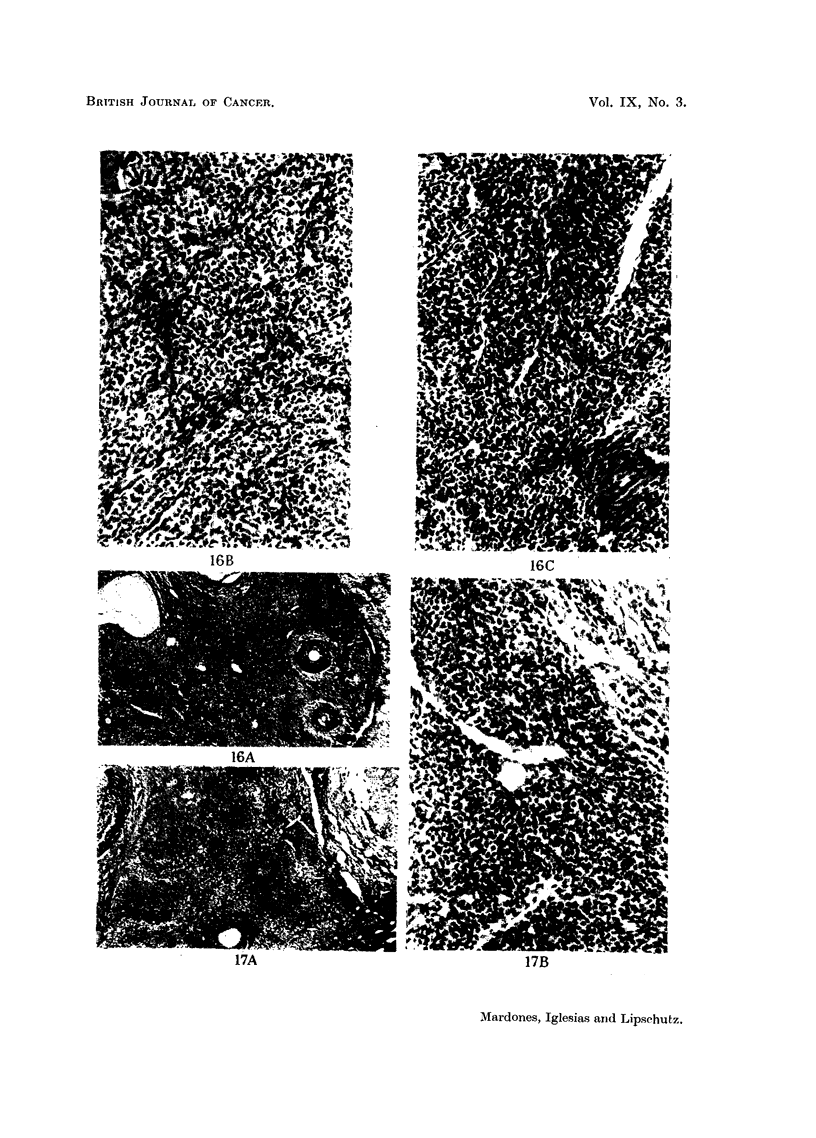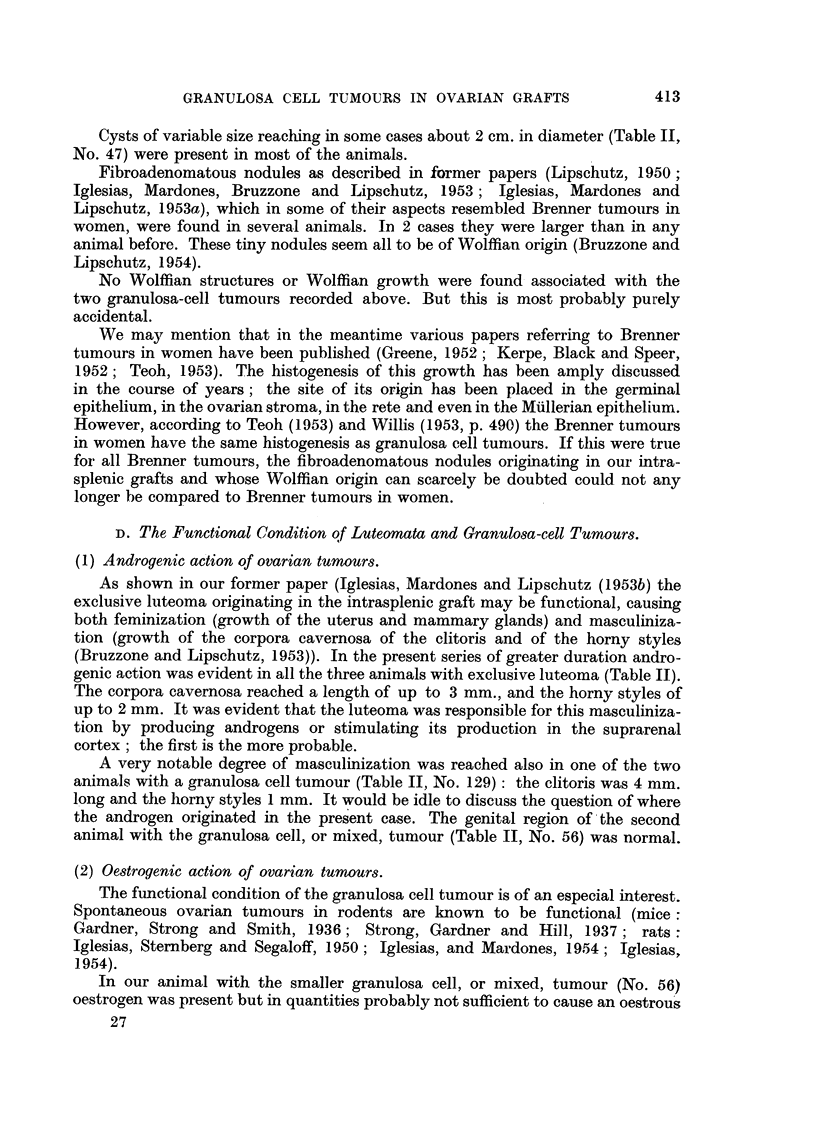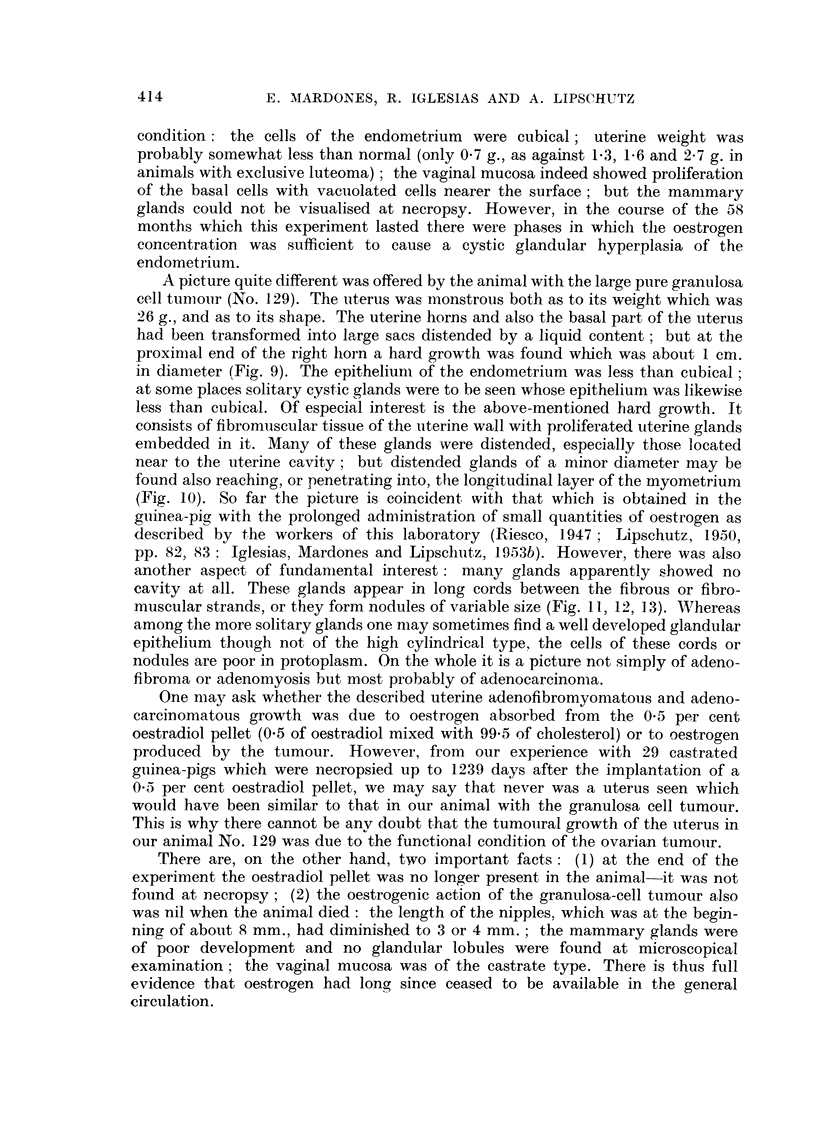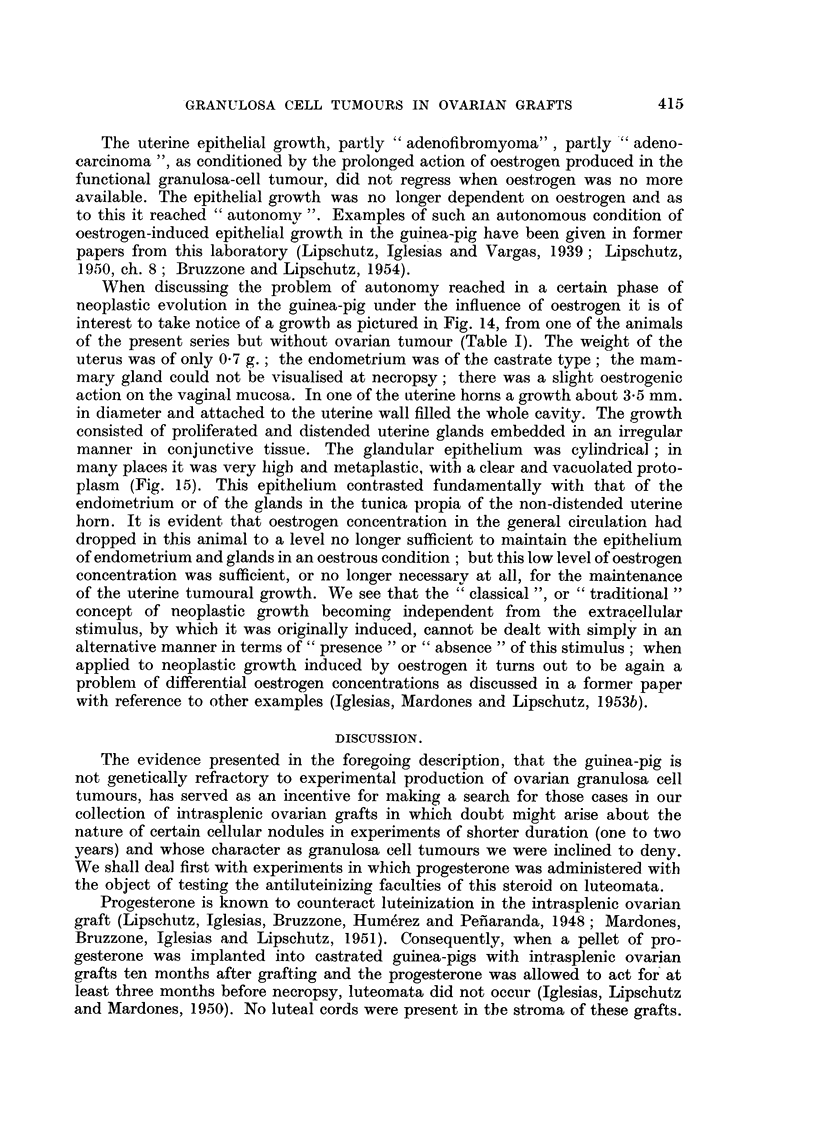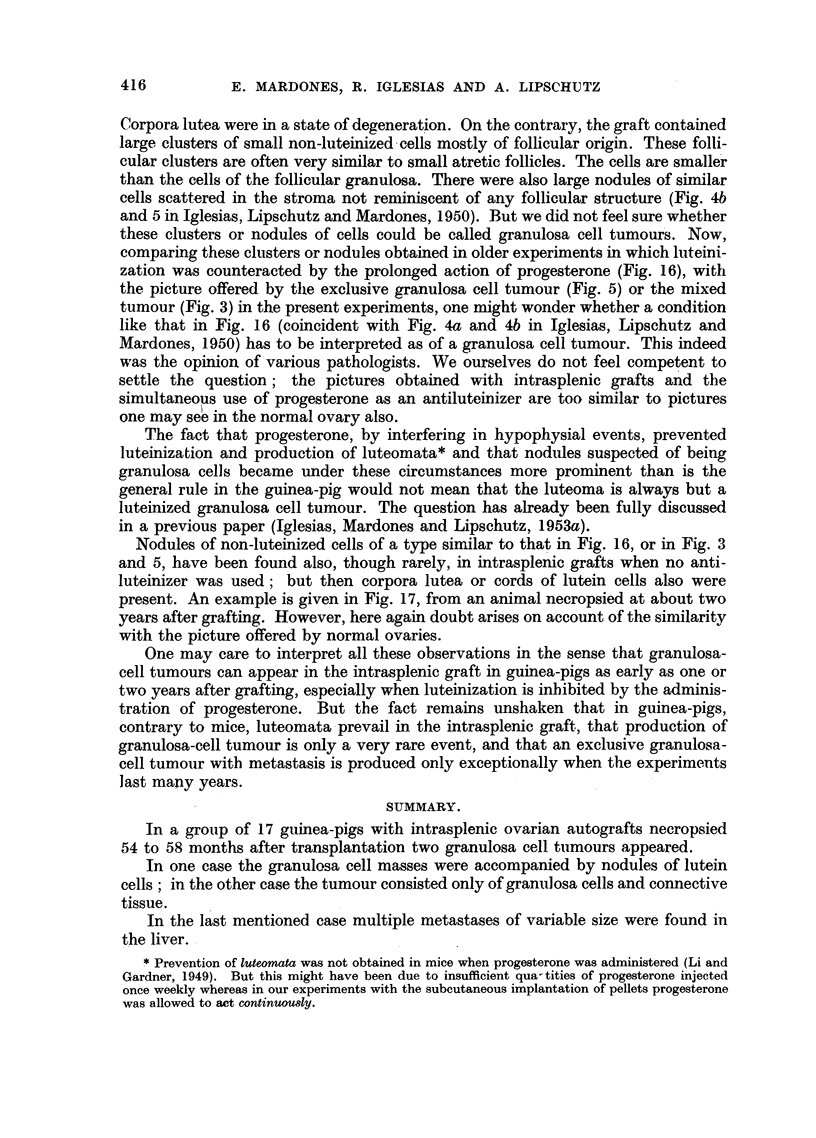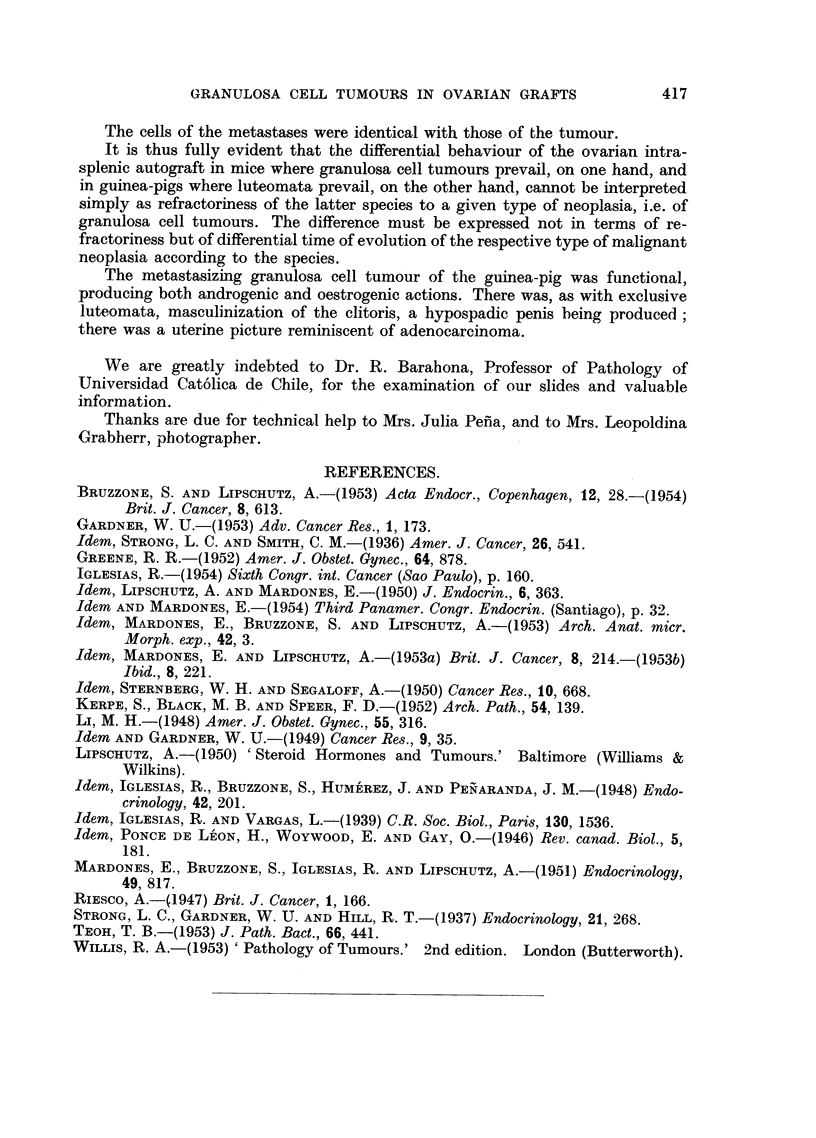# Granulosa Cell Tumours in Intrasplenic Ovarian Grafts, with Intrahepatic Metastases, in Guinea-pigs at Five Years after Grafting

**DOI:** 10.1038/bjc.1955.39

**Published:** 1955-09

**Authors:** E. Mardones, R. Iglesias, A. Lipschutz

## Abstract

**Images:**


### GRANULOSA CELL TUMOURS IN INTRASPLENIC OVARIAN GRAFTS, WITH INTRAHEPATIC METASTASES, IN GUINEA-PIGS AT FIVE YEARS AFTER GRAFTING.

E. MARDONES, R. IGLESIAS AND A. LIPSCHUTZ.

*From Instituto de Medicina Experimental, Servicio Nacional de Salud, Avenida Irarrázaval* 849, *Santiago de Chile.*

Received for publication June 11, 1955.

EVIDENCE has been given in previous papers (Lipschutz, Ponce de León, Woywood and Gay, 1946 ; Iglesias, Mardones and Lipschutz, 1953*a*) that luteomata develop in intrasplenic autografts in the castrated guinea-pig ; in experiments lasting 10 to 36 months these luteomata reached an incidence of about 60 per cent. They may replace the ovary completely (" exclusive " luteoma) ; they attain sometimes an enormous size. They infiltrate the spleen. They are not transplantable. On the contrary, in other rodents granulosa cell tumours prevail as shown as early as 1944 by the work of Biskind and others in the rat, by Li, Gardner, Furth and others in mice (Gardner, 1953 ; also still unpublished work of Mardones). In mice metastases may occur though rarely (Li, 1948).

Indeed, one may find in intrasplenic grafts in guinea-pigs nodules or cords of non-luteinized cells deriving from follicles or from the stroma ; similar nodules or cords may constitute an important part of the latter. But we did not feel sure whether these nodules deserve the name of a thecoma or granulosa cell tumour. Though so many massive exclusive luteomata developed in our experiments when continued for as long as three years, till recently we had never seen massive or exclusive granulosa cell tumours in guinea-pigs and we never had seen metastasis.

It seemed reasonable to ask (Iglesias, Lipschutz and Mardones, 1950) whether the differential behaviour of the various rodents had to be explained as due to a difference of the time necessary for the induction of metastasizing ovarian tumours according to the species. However, since there was no experimental evidence of this, we became rather inclined to interpret the mentioned differential behaviour as an example of a genetical refractoriness to a certain type of neoplasia. But when examining lately a series of guinea-pigs necropsied almost five years after grafting the ovary into the spleen, we found an exclusive and metastasizing granulosa-cell tumour and a mixed luteoma-granulosa-cell tumour. This finding gives for the first time full evidence that the genetical difference as to neoplastic growth in the given species must be expressed not in terms of refractoriness but of differential time of evolution of the respective type of neoplasia. Needless to say that this opens a host of fundamental problems of evolutional tumourigenic dynamics in general. These two cases of granulosa cell tumours shall be described in the present paper.

The animals belonged to a series in which pellets containing very small amounts of oestradiol were implanted with the purpose of studying quantitative aspects of the inhibitory action of oestrogen on intrasplenic ovarian tumourigenesis, in continuation of former work in this field (Iglesias, Mardones and Lipschutz,

1953*b*). However, the long duration of the present experiments makes it unnecessary to deal here with this special aspect. We have found (unpublished work) that oestrogenic action of similar pellets in castrated guinea-pigs diminishes in time. The vagina which had opened closes again ; the nipples which had grown diminish in size. We do not yet know why oestrogenic action ceases ; but at about two years after implantation of the pellet containing but 0·1 to 3·0 per cent of oestradiol mixed with cholesterol, diminution of oestrogenic action on the vagina and nipples was general. It is reasonable to suppose that the control of the hypophysis by the circulating oestrogen also relaxes at that time and that the gorgeous evolution of luteomatous growth and of granulosa cell tumours in our animals took place in the course of the additional two to three years when production or delivery of gonadotrophic hormones was no more under the control of oestrogen. But both the incidence of luteomata, which was smaller than without oestrogen, and the smaller size of some of these growths apparently still denounce some inhibitory action of oestrogen in these experiments even when lasting as much as almost five years.

### RESULTS.

### A. *Incidence and Description of Tumours.*

The material is summarized in Tables I and II.

There were tumours in 7 out of 17 animals. The incidence is somewhat smaller than in our former series without oestrogen (Table I in Iglesias, Mardones and Lipschutz, 1953*a*). There were 3 exclusive luteomata (No. 232, 145, 47) ; no follicular structures and no individual corpora lutea were present besides the masses of lutein cells. The other two luteomata were of rather smaller size, especially No. 116 ; but even in this case luteomatous cords were infiltrating the spleen.

### B. *Granulosa Cell Tumours.*

As mentioned there were two cases with granulosa cell tumours.

(1) (Table II, No. 56). The greatest diameter of the tumour was less than 1 cm. Already at low augmentation (Fig. 1) two different kinds of nodules could be distinguished : clear and dark ones, in general separated from one another by fibrous strands. Part of the clear nodules are composed of large cells with a vacuolated protoplasm. These cells are coincident, as to structure, with those of the corpus luteum. The darker nodules are constituted of smaller cells of the granulosa cell type. The growth is thus a mixed tumour consisting both of luteomatous and granulosa cell tissue (Fig. 2). However, granulosa cell tissue greatly prevailed (Fig. 3) : probably no less than four-fifths, or more, of the tumour were nodules or large areas of the granulosa-cell type. No follicular structures were present ; there were indeed in some granulosa cell nodules small cavities which one may suspect of being the remnants of follicular structures. In some areas the granulosa cells evidently underwent a transformation ; their protoplasm became vacuolated, and several of the clear nodules in Fig. 1 consist of these vacuolated granulosa cells. But it is remarkable that the vacuolated granulosa cells did not take the aspect of lutein cells ; there was a definite difference between both even when vacuolated. In some places nodules of granulosa cells are in immediate contact with lutein cells. A nodule of granulosa cells may even be completely surrounded by lutein cells.

TABLE I.—*Seventeen Castrated Guinea-pigs with Intrasplenic Ovarian Grafts.*

| Duration of experiment. Months. | Number of animals. Total. | Animals with: HF. | Luteoma. | Granulosa-cell tumour. |
|---|---|---|---|---|
| 56–58* | 17 | 6 | 5† | 2‡ |

* One animal only 48 months : No. 232 in Table II.
† Out of these, 3 were exclusive luteomata : No. 232, 145, 47 in Table II.
‡ One of these was a mixed tumour : No. 56 in Table II.

TABLE II.—*Description of 7 Tumours and of their Functional Condition.*

| CLI. No. | Duration (days). | Ovarian tumours (mm.). | Genital region: Clitoris. Corpora cavernosa (mm.). | Horny styles (mm.). | Uterine weight (g.). | Uterine epithelium. | Mammary gland. |
|---|---|---|---|---|---|---|---|
| 232 | 1441 | Exclusive luteoma 13 × 8 | 3 | 1·5 | 1·3 | Metaplasia Cystic hyperplasia | — |
| 145 | 1683 | Exclusive luteoma 10 × 5 Large " Brenner " 4 × 2 | 2 | 2 | 1·6 | Metaplasia Cystic hyperplasia Polyps | Pregn. |
| 47 | 1764 | Exclusive luteoma 13 × 8 | 3 | 2 | 2·7 | Metaplasia Cystic hyperplasia Polyps | Pregn |
| 116 | 1681 | Luteoma, infiltrative 5 × 3 | — | — | 1·3 | Cylindrical epithelium | — |
| 61 | 1749 | Luteoma, infiltrative 8 × 6 | — | — | 1·0 | Cubical and cylindrical epithelium Cystic hyperplasia | 0 |
| 56 | 1765 | Mixed : luteoma *granulosa cell* 7 × 6 | — | — | 0·7 | Cubical epithelium Cystic hyperplasia | — |
| 129 | 1666 | *Granulosa cell* Fig. 4 | 4 | 1 | 26·0 | " Adenocarcinoma " see text and Fig. 11–13 | 0 |

In right-hand column — = glandular tissue not found at necropsy and not examined microscopically ; 0 = glandular tissue not found at necropsy and neither at microscopical examination.

(2) (Table II, No. 129). A formidable growth was found at necropsy in the splenic region (Fig. 4). The weight of the spleen together with the growth was 51 g., of which only about 1 g. corresponds to the spleen. At incision compact tissue was seen intermingled with what seemed to be necrotic and haemorrhagic masses. The colour of the tumour was reddish-grey. Irregularities of the same colour were to be seen on the surface of the liver ; it was the same when incisions were made. At microscopical examination the growth was shown to consist of compact masses of cells of the granulosa type (Fig. 5) adjoining necrotic or haemorrhagic zones, or zones of conjunctive tissue, sometimes fibrous but more often loose and oedematous. At many places the granulosa cells are intermingled with a variable number of small cells, probably leucocytes. The liver was filled with nodules which were an exact replica of the tumour (Fig. 6, 7); the microphotographs of both were identical (Fig. 6 and 8). There was not the slightest doubt about the intrahepatic nodules being metastases of the tumour

### C. *Growth of Wolffian structures.*

As in former work with intrasplenic ovarian autografts in castrated guinea-pigs atypical growth of Wolffian structures was very prominent also in the present series of long duration.

---

### EXPLANATION OF PLATES.

*Fig.* 1, 2, 3, *are from the same animal.*

FIG. 1.—Intrasplenic ovarian graft; 1765 days (CLI.56). No follicular structures and no individual corpora lutea. The tumour consists of luteomatous nodules and of nodules of granulosa cells. The latter prevail. × 4.

FIG. 2.—Mixed part of the tumour. At the bottom, cords of luteal cells separated from the spleen by a fibrous capsule. At the top luteomatous cords in immediate contact with large nodule of granulosa cells. × 100.

FIG. 3.—Nodule of granulosa cells. Circular disposition of the cells at various places. × 300.

*Figs.* 4 *to* 8 *are of tumour and metastases of the same animal.*

FIG. 4.—Intrasplenic ovarian graft; 1666 days (CLI. 129). Enormous tumour; to the left adhesion of epiploon. × 4/5.

FIG. 5.—Tumour; compact mass of granulosa cells. × 200.

FIG. 6.—Large intrahepatic metastasis. × 15.

FIG. 7.—Small intrahepatic metastasis. × 100.

FIG. 8.—Compact mass of granulosa cells of large metastasis. Many leucocytes. × 200.

*Fig.* 9 *to* 13 *are from the uterus of the same animal as Fig.* 4 *to* 8.

FIG. 9.—Large uterine nodule. Note unilateral position of the latter which is typical of the oestrogen-induced nodule. Cystic enlargement of glands filling the uterine cavity. × 4.

FIG. 10.—Fibromyoadenomatous part of the nodule. Enlarged glands in the longitudinal layer and reaching the subserous layer of fibromuscular tissue which can be seen in uteri under the prolonged action of oestrogen. × 70.

FIGS. 11, 12 and 13.—Epithelial cords in the fibromyoadenomatous nodule. "Adenocarcinoma." × 100.

*Fig.* 14 *and* 15 *are of the same animal.*

FIG. 14.—A. Distended uterine wall surrounding fibroadenomatous polyps. Note the irregular disposition of the distended glands.

B. Uterus of the same animal; uterus not distended. Intrasplenic ovarian graft, 1762 days (CLI. 50). There was no ovarian tumour, though indeed cords of luteinized cells, an haemorrhagic follicle, Graafian follicles and corpora lutea were present. The small uterine weight, the castrate condition of the endometrium and the undeveloped mammary gland make it clear that the fibroadenomatous polyp was produced long before necropsy. × 16.

FIG. 15.—The same polyp as in Fig. 14A. Note metaplasia of endometrium and glands. × 100.

FIG. 16.—A. Ovarian intrasplenic graft, 360 days after grafting (CXXVI. 96). The last 90 days progesterone was administered by absorption from pellet. A considerable part of the graft is occupied by "follicular clusters." Granulosa cell tumour? × 35.

B and C. Details of the same. × 200.

FIG. 17.—A. Ovarian intrasplenic graft, 798 days after grafting (CLI. 68). A considerable part of the ovary is occupied by "follicular clusters." Granulosa cell tumour? A pellet containing 1 per cent of oestrogen was implanted. No luteoma was present. × 35.

B. Details of the same. × 200.

1

2

3

4

5

6

7

9

10

8

Mardones, Iglesias and Lipschutz.

11

12

13

14B

14A

15

16B

16C

16A

17A

17B

Mardones, Iglesias and Lipschutz.

Cysts of variable size reaching in some cases about 2 cm. in diameter (Table II, No. 47) were present in most of the animals.

Fibroadenomatous nodules as described in former papers (Lipschutz, 1950; Iglesias, Mardones, Bruzzone and Lipschutz, 1953; Iglesias, Mardones and Lipschutz, 1953*a*), which in some of their aspects resembled Brenner tumours in women, were found in several animals. In 2 cases they were larger than in any animal before. These tiny nodules seem all to be of Wolffian origin (Bruzzone and Lipschutz, 1954).

No Wolffian structures or Wolffian growth were found associated with the two granulosa-cell tumours recorded above. But this is most probably purely accidental.

We may mention that in the meantime various papers referring to Brenner tumours in women have been published (Greene, 1952; Kerpe, Black and Speer, 1952; Teoh, 1953). The histogenesis of this growth has been amply discussed in the course of years; the site of its origin has been placed in the germinal epithelium, in the ovarian stroma, in the rete and even in the Müllerian epithelium. However, according to Teoh (1953) and Willis (1953, p. 490) the Brenner tumours in women have the same histogenesis as granulosa cell tumours. If this were true for all Brenner tumours, the fibroadenomatous nodules originating in our intrasplenic grafts and whose Wolffian origin can scarcely be doubted could not any longer be compared to Brenner tumours in women.

### D. *The Functional Condition of Luteomata and Granulosa-cell Tumours.*

(1) *Androgenic action of ovarian tumours.*

As shown in our former paper (Iglesias, Mardones and Lipschutz (1953*b*) the exclusive luteoma originating in the intrasplenic graft may be functional, causing both feminization (growth of the uterus and mammary glands) and masculinization (growth of the corpora cavernosa of the clitoris and of the horny styles (Bruzzone and Lipschutz, 1953)). In the present series of greater duration androgenic action was evident in all the three animals with exclusive luteoma (Table II). The corpora cavernosa reached a length of up to 3 mm., and the horny styles of up to 2 mm. It was evident that the luteoma was responsible for this masculinization by producing androgens or stimulating its production in the suprarenal cortex; the first is the more probable.

A very notable degree of masculinization was reached also in one of the two animals with a granulosa cell tumour (Table II, No. 129): the clitoris was 4 mm. long and the horny styles 1 mm. It would be idle to discuss the question of where the androgen originated in the present case. The genital region of the second animal with the granulosa cell, or mixed, tumour (Table II, No. 56) was normal.

(2) *Oestrogenic action of ovarian tumours.*

The functional condition of the granulosa cell tumour is of an especial interest. Spontaneous ovarian tumours in rodents are known to be functional (mice: Gardner, Strong and Smith, 1936; Strong, Gardner and Hill, 1937; rats: Iglesias, Sternberg and Segaloff, 1950; Iglesias, and Mardones, 1954; Iglesias, 1954).

In our animal with the smaller granulosa cell, or mixed, tumour (No. 56) oestrogen was present but in quantities probably not sufficient to cause an oestrous condition: the cells of the endometrium were cubical; uterine weight was probably somewhat less than normal (only 0·7 g., as against 1·3, 1·6 and 2·7 g. in animals with exclusive luteoma); the vaginal mucosa indeed showed proliferation of the basal cells with vacuolated cells nearer the surface; but the mammary glands could not be visualised at necropsy. However, in the course of the 58 months which this experiment lasted there were phases in which the oestrogen concentration was sufficient to cause a cystic glandular hyperplasia of the endometrium.

A picture quite different was offered by the animal with the large pure granulosa cell tumour (No. 129). The uterus was monstrous both as to its weight which was 26 g., and as to its shape. The uterine horns and also the basal part of the uterus had been transformed into large sacs distended by a liquid content; but at the proximal end of the right horn a hard growth was found which was about 1 cm. in diameter (Fig. 9). The epithelium of the endometrium was less than cubical; at some places solitary cystic glands were to be seen whose epithelium was likewise less than cubical. Of especial interest is the above-mentioned hard growth. It consists of fibromuscular tissue of the uterine wall with proliferated uterine glands embedded in it. Many of these glands were distended, especially those located near to the uterine cavity; but distended glands of a minor diameter may be found also reaching, or penetrating into, the longitudinal layer of the myometrium (Fig. 10). So far the picture is coincident with that which is obtained in the guinea-pig with the prolonged administration of small quantities of oestrogen as described by the workers of this laboratory (Riesco, 1947; Lipschutz, 1950, pp. 82, 83; Iglesias, Mardones and Lipschutz, 1953*b*). However, there was also another aspect of fundamental interest: many glands apparently showed no cavity at all. These glands appear in long cords between the fibrous or fibromuscular strands, or they form nodules of variable size (Fig. 11, 12, 13). Whereas among the more solitary glands one may sometimes find a well developed glandular epithelium though not of the high cylindrical type, the cells of these cords or nodules are poor in protoplasm. On the whole it is a picture not simply of adenofibroma or adenomyosis but most probably of adenocarcinoma.

One may ask whether the described uterine adenofibromyomatous and adenocarcinomatous growth was due to oestrogen absorbed from the 0·5 per cent oestradiol pellet (0·5 of oestradiol mixed with 99·5 of cholesterol) or to oestrogen produced by the tumour. However, from our experience with 29 castrated guinea-pigs which were necropsied up to 1239 days after the implantation of a 0·5 per cent oestradiol pellet, we may say that never was a uterus seen which would have been similar to that in our animal with the granulosa cell tumour. This is why there cannot be any doubt that the tumoural growth of the uterus in our animal No. 129 was due to the functional condition of the ovarian tumour.

There are, on the other hand, two important facts: (1) at the end of the experiment the oestradiol pellet was no longer present in the animal—it was not found at necropsy; (2) the oestrogenic action of the granulosa-cell tumour also was nil when the animal died: the length of the nipples, which was at the beginning of about 8 mm., had diminished to 3 or 4 mm.; the mammary glands were of poor development and no glandular lobules were found at microscopical examination; the vaginal mucosa was of the castrate type. There is thus full evidence that oestrogen had long since ceased to be available in the general circulation.

The uterine epithelial growth, partly " adenofibromyoma", partly " adenocarcinoma ", as conditioned by the prolonged action of oestrogen produced in the functional granulosa-cell tumour, did not regress when oestrogen was no more available. The epithelial growth was no longer dependent on oestrogen and as to this it reached " autonomy ". Examples of such an autonomous condition of oestrogen-induced epithelial growth in the guinea-pig have been given in former papers from this laboratory (Lipschutz, Iglesias and Vargas, 1939 ; Lipschutz, 1950, ch. 8 ; Bruzzone and Lipschutz, 1954).

When discussing the problem of autonomy reached in a certain phase of neoplastic evolution in the guinea-pig under the influence of oestrogen it is of interest to take notice of a growth as pictured in Fig. 14, from one of the animals of the present series but without ovarian tumour (Table I). The weight of the uterus was of only 0·7 g. ; the endometrium was of the castrate type ; the mammary gland could not be visualised at necropsy ; there was a slight oestrogenic action on the vaginal mucosa. In one of the uterine horns a growth about 3·5 mm. in diameter and attached to the uterine wall filled the whole cavity. The growth consisted of proliferated and distended uterine glands embedded in an irregular manner in conjunctive tissue. The glandular epithelium was cylindrical ; in many places it was very high and metaplastic, with a clear and vacuolated protoplasm (Fig. 15). This epithelium contrasted fundamentally with that of the endometrium or of the glands in the tunica propia of the non-distended uterine horn. It is evident that oestrogen concentration in the general circulation had dropped in this animal to a level no longer sufficient to maintain the epithelium of endometrium and glands in an oestrous condition ; but this low level of oestrogen concentration was sufficient, or no longer necessary at all, for the maintenance of the uterine tumoural growth. We see that the " classical ", or " traditional " concept of neoplastic growth becoming independent from the extracellular stimulus, by which it was originally induced, cannot be dealt with simply in an alternative manner in terms of " presence " or " absence " of this stimulus ; when applied to neoplastic growth induced by oestrogen it turns out to be again a problem of differential oestrogen concentrations as discussed in a former paper with reference to other examples (Iglesias, Mardones and Lipschutz, 1953*b*).

### DISCUSSION.

The evidence presented in the foregoing description, that the guinea-pig is not genetically refractory to experimental production of ovarian granulosa cell tumours, has served as an incentive for making a search for those cases in our collection of intrasplenic ovarian grafts in which doubt might arise about the nature of certain cellular nodules in experiments of shorter duration (one to two years) and whose character as granulosa cell tumours we were inclined to deny. We shall deal first with experiments in which progesterone was administered with the object of testing the antiluteinizing faculties of this steroid on luteomata.

Progesterone is known to counteract luteinization in the intrasplenic ovarian graft (Lipschutz, Iglesias, Bruzzone, Humérez and Peñaranda, 1948 ; Mardones, Bruzzone, Iglesias and Lipschutz, 1951). Consequently, when a pellet of progesterone was implanted into castrated guinea-pigs with intrasplenic ovarian grafts ten months after grafting and the progesterone was allowed to act for at least three months before necropsy, luteomata did not occur (Iglesias, Lipschutz and Mardones, 1950). No luteal cords were present in the stroma of these grafts.

Corpora lutea were in a state of degeneration. On the contrary, the graft contained large clusters of small non-luteinized cells mostly of follicular origin. These follicular clusters are often very similar to small atretic follicles. The cells are smaller than the cells of the follicular granulosa. There were also large nodules of similar cells scattered in the stroma not reminiscent of any follicular structure (Fig. 4*b* and 5 in Iglesias, Lipschutz and Mardones, 1950). But we did not feel sure whether these clusters or nodules of cells could be called granulosa cell tumours. Now, comparing these clusters or nodules obtained in older experiments in which luteinization was counteracted by the prolonged action of progesterone (Fig. 16), with the picture offered by the exclusive granulosa cell tumour (Fig. 5) or the mixed tumour (Fig. 3) in the present experiments, one might wonder whether a condition like that in Fig. 16 (coincident with Fig. 4*a* and 4*b* in Iglesias, Lipschutz and Mardones, 1950) has to be interpreted as of a granulosa cell tumour. This indeed was the opinion of various pathologists. We ourselves do not feel competent to settle the question; the pictures obtained with intrasplenic grafts and the simultaneous use of progesterone as an antiluteinizer are too similar to pictures one may see in the normal ovary also.

The fact that progesterone, by interfering in hypophysial events, prevented luteinization and production of luteomata* and that nodules suspected of being granulosa cells became under these circumstances more prominent than is the general rule in the guinea-pig would not mean that the luteoma is always but a luteinized granulosa cell tumour. The question has already been fully discussed in a previous paper (Iglesias, Mardones and Lipschutz, 1953*a*).

Nodules of non-luteinized cells of a type similar to that in Fig. 16, or in Fig. 3 and 5, have been found also, though rarely, in intrasplenic grafts when no antiluteinizer was used; but then corpora lutea or cords of lutein cells also were present. An example is given in Fig. 17, from an animal necropsied at about two years after grafting. However, here again doubt arises on account of the similarity with the picture offered by normal ovaries.

One may care to interpret all these observations in the sense that granulosa-cell tumours can appear in the intrasplenic graft in guinea-pigs as early as one or two years after grafting, especially when luteinization is inhibited by the administration of progesterone. But the fact remains unshaken that in guinea-pigs, contrary to mice, luteomata prevail in the intrasplenic graft, that production of granulosa-cell tumour is only a very rare event, and that an exclusive granulosa-cell tumour with metastasis is produced only exceptionally when the experiments last many years.

### SUMMARY.

In a group of 17 guinea-pigs with intrasplenic ovarian autografts necropsied 54 to 58 months after transplantation two granulosa cell tumours appeared.

In one case the granulosa cell masses were accompanied by nodules of lutein cells; in the other case the tumour consisted only of granulosa cells and connective tissue.

In the last mentioned case multiple metastases of variable size were found in the liver.

* Prevention of *luteomata* was not obtained in mice when progesterone was administered (Li and Gardner, 1949). But this might have been due to insufficient quantities of progesterone injected once weekly whereas in our experiments with the subcutaneous implantation of pellets progesterone was allowed to act *continuously*.

The cells of the metastases were identical with those of the tumour.

It is thus fully evident that the differential behaviour of the ovarian intrasplenic autograft in mice where granulosa cell tumours prevail, on one hand, and in guinea-pigs where luteomata prevail, on the other hand, cannot be interpreted simply as refractoriness of the latter species to a given type of neoplasia, i.e. of granulosa cell tumours. The difference must be expressed not in terms of refractoriness but of differential time of evolution of the respective type of malignant neoplasia according to the species.

The metastasizing granulosa cell tumour of the guinea-pig was functional, producing both androgenic and oestrogenic actions. There was, as with exclusive luteomata, masculinization of the clitoris, a hypospadic penis being produced ; there was a uterine picture reminiscent of adenocarcinoma.

We are greatly indebted to Dr. R. Barahona, Professor of Pathology of Universidad Católica de Chile, for the examination of our slides and valuable information.

Thanks are due for technical help to Mrs. Julia Peña, and to Mrs. Leopoldina Grabherr, photographer.

### REFERENCES.

BRUZZONE, S. AND LIPSCHUTZ, A.—(1953) *Acta Endocr., Copenhagen*, **12**, 28.—(1954) *Brit. J. Cancer*, **8**, 613.
GARDNER, W. U.—(1953) *Adv. Cancer Res.*, **1**, 173.
*Idem*, STRONG, L. C. AND SMITH, C. M.—(1936) *Amer. J. Cancer*, **26**, 541.
GREENE, R. R.—(1952) *Amer. J. Obstet. Gynec.*, **64**, 878.
IGLESIAS, R.—(1954) *Sixth Congr. int. Cancer* (*Sao Paulo*), p. 160.
*Idem*, LIPSCHUTZ, A. AND MARDONES, E.—(1950) *J. Endocrin.*, **6**, 363.
*Idem* AND MARDONES, E.—(1954) *Third Panamer. Congr. Endocrin.* (Santiago), p. 32.
*Idem*, MARDONES, E., BRUZZONE, S. AND LIPSCHUTZ, A.—(1953) *Arch. Anat. micr. Morph. exp.*, **42**, 3.
*Idem*, MARDONES, E. AND LIPSCHUTZ, A.—(1953*a*) *Brit. J. Cancer*, **8**, 214.—(1953*b*) *Ibid.*, **8**, 221.
*Idem*, STERNBERG, W. H. AND SEGALOFF, A.—(1950) *Cancer Res.*, **10**, 668.
KERPE, S., BLACK, M. B. AND SPEER, F. D.—(1952) *Arch. Path.*, **54**, 139.
LI, M. H.—(1948) *Amer. J. Obstet. Gynec.*, **55**, 316.
*Idem* AND GARDNER, W. U.—(1949) *Cancer Res.*, **9**, 35.
LIPSCHUTZ, A.—(1950) 'Steroid Hormones and Tumours.' Baltimore (Williams & Wilkins).
*Idem*, IGLESIAS, R., BRUZZONE, S., HUMÉREZ, J. AND PEÑARANDA, J. M.—(1948) *Endocrinology*, **42**, 201.
*Idem*, IGLESIAS, R. AND VARGAS, L.—(1939) *C.R. Soc. Biol., Paris*, **130**, 1536.
*Idem*, PONCE DE LÉON, H., WOYWOOD, E. AND GAY, O.—(1946) *Rev. canad. Biol.*, **5**, 181.
MARDONES, E., BRUZZONE, S., IGLESIAS, R. AND LIPSCHUTZ, A.—(1951) *Endocrinology*, **49**, 817.
RIESCO, A.—(1947) *Brit. J. Cancer*, **1**, 166.
STRONG, L. C., GARDNER, W. U. AND HILL, R. T.—(1937) *Endocrinology*, **21**, 268.
TEOH, T. B.—(1953) *J. Path. Bact.*, **66**, 441.
WILLIS, R. A.—(1953) 'Pathology of Tumours.' 2nd edition. London (Butterworth).